# Dominant negative mutant Cyclin T1 proteins inhibit HIV transcription by specifically degrading Tat

**DOI:** 10.1186/1742-4690-5-63

**Published:** 2008-07-11

**Authors:** Julie K Jadlowsky, Masanori Nojima, Antje Schulte, Matthias Geyer, Takashi Okamoto, Koh Fujinaga

**Affiliations:** 1 Division of Infectious Diseases, Department of Medicine, Department of Molecular Biology and Microbiology, Case Western Reserve University School of Medicine, Cleveland, Ohio, USA; 2 Max-Planck-Institut für molekulare Physiologie, Abteilung Physikalische Biochemie, Dortmund, Germany; 3 Department of Molecular and Cellular Biology, Nagoya City University Graduate School of Medical Sciences, Nagoya, Japan

## Abstract

**Background:**

The positive transcription elongation factor b (P-TEFb) is an essential cellular co-factor for the transcription of the human immunodeficiency virus type 1 (HIV-1). The cyclin T1 (CycT1) subunit of P-TEFb associates with a viral protein, Tat, at the transactivation response element (TAR). This represents a critical and necessary step for the stimulation of transcriptional elongation. Therefore, CycT1 may serve as a potential target for the development of anti-HIV therapies.

**Results:**

To create effective inhibitors of HIV transcription, mutant CycT1 proteins were constructed based upon sequence similarities between CycT1 and other cyclin molecules, as well as the defined crystal structure of CycT1. One of these mutants, termed CycT1-U7, showed a potent dominant negative effect on Tat-dependent HIV transcription despite a remarkably low steady-state expression level. Surprisingly, the expression levels of Tat proteins co-expressed with CycT1-U7 were significantly lower than Tat co-expressed with wild type CycT1. However, the expression levels of CycT1-U7 and Tat were restored by treatment with proteasome inhibitors. Concomitantly, the dominant negative effect of CycT1-U7 was abolished by these inhibitors.

**Conclusion:**

These results suggest that CycT1-U7 inhibits HIV transcription by promoting a rapid degradation of Tat. These mutant CycT1 proteins represent a novel class of specific inhibitors for HIV transcription that could potentially be used in the design of anti-viral therapy.

## Background

The transcription of human immunodeficiency virus type 1 (HIV-1) is a highly regulated process in which several host cellular co-factors and the viral transactivator protein Tat are involved [1,2]. Tat stimulates the elongation of transcription with the aid of the positive transcription elongation factor b (P-TEFb), a heterodimer comprised of cyclin T1 (CycT1) and cyclin dependent kinase 9 (Cdk9). Tat and CycT1 bind to the transactivation response element (TAR), an RNA stem loop structure located at the 5'-end (+1 to +59) of all viral transcripts [3-5]. This interaction results in the recruitment of Cdk9 and the subsequent stimulation of its kinase activity by Tat [6]. Among three distinct P-TEFb complexes (CycT1/Cdk9, CycT2/Cdk9, and CycK/Cdk9), only the CycT1/Cdk9 complex can support Tat transactivation [7-9].

The interaction between Tat, TAR, and CycT1 has been extensively studied [2-5,8,10]. Tat binds to the bulge region (+23 to +25) of TAR and the CycT1 subunit of P-TEFb through its central arginine-rich motif (ARM; a.a. 49–60) and its N-terminal activation domain (a.a. 1–48), respectively. CycT1, in turn, is thought to bind to the central loop (+30 to +35) of TAR through its Tat-TAR recognition motif (TRM; a.a. 251–271) in the presence of Tat [1,2]. Human CycT1 is comprised of 726 amino acids and contains a cyclin box repeat domain (from positions 31 to 250), a coiled-coil sequence (from positions 379 to 530), and a PEST sequence (from positions 709 to 726). The N-terminal cyclin boxes are important for binding and activation of Cdk9. Residues from positions 251 to 272 are essential for the zinc ion-mediated binding between Tat and TAR [5]. This region also interacts with the HEXIM1 protein and 7SK small nuclear RNA, which negatively regulate the kinase activity of P-TEFb [11-15]. The C-terminal region (a.a. 273–726) of CycT1 is dispensable for Tat transactivation since the N-terminal cyclin repeats (a.a. 1–250) and TRM (a.a. 251–272) of CycT1 interact with Cdk9, Tat and TAR [3-5,9,16,17]. Recently, we have determined the crystal structure of the N-terminal region (a.a. 1–280) of human CycT1 [18] and its interacting dimeric Cyclin T-binding domain in HEXIM1 [19].

Since P-TEFb is the essential cellular host co-factor of the viral Tat protein, this interaction serves as a potential target for anti-HIV therapeutics. Several approaches have been taken to block HIV transcription by targeting P-TEFb. First, mutant Cdk9 proteins defective in kinase activity have been shown to inhibit HIV transcription in cell culture systems [20]. A number of small compounds that inhibit Cdk9 activities or disrupt the Tat/TAR/P-TEFb interaction have also been tested [20-28]. Another approach by Napolitano et al. aimed to inactivate Cdk9 by an oligomerization chain reaction [29]. Additionally, our group has constructed chimeric proteins containing wild type (wt) CycT1 and mutant Cdk9 which inhibited HIV replication up to 90% [30]. Moreover, several CycT1-binding proteins and their truncation mutants have been used as inhibitors of Tat transactivation [31-33]. Finally, Bai et al. demonstrated that intrabodies against CycT1 inhibited Tat stimulated transactivation [34]. It is important to note, however, that because P-TEFb is involved in the transcription of many cellular genes [35], it is critical to exclusively block HIV-specific pathways in order to develop safe and effective anti-HIV therapies.

In this study, we sought to construct dominant negative CycT1 mutant proteins capable of blocking HIV transcription. A sequence alignment between the cyclin proteins CycT1, T2 and K revealed ten very well-conserved regions that are essential for the formation of the alpha-helical cyclin box repeat domain. We introduced random mutations in the nine most conserved amino acid clusters in these regions and tested the resulting mutant CycT1 proteins for their ability to block HIV transcription. One of the mutant proteins, called CycT1-U7, showed a potent, yet specific, dominant negative effect on HIV transcription, although the steady-state expression level of CycT1-U7 was remarkably low. Western blot analysis indicated that the expression level of the Tat proteins co-expressed with CycT1-U7 was also significantly lower than those co-expressed with wt CycT1. Proteasome inhibitors restored the expression of CycT1-U7 and Tat proteins. As a consequence, these inhibitors diminished the dominant negative effect elicited by over-expression of CycT1-U7. Our results suggest that CycT1-U7 inhibits HIV transcription by promoting a rapid degradation of Tat proteins. These mutant CycT1 proteins represent a novel class of specific inhibitors for HIV transcription, which might be further utilized in development of safe and effective anti-HIV therapies.

## Results

### Construction and screening of CycT1 mutants

**Figure 1:**
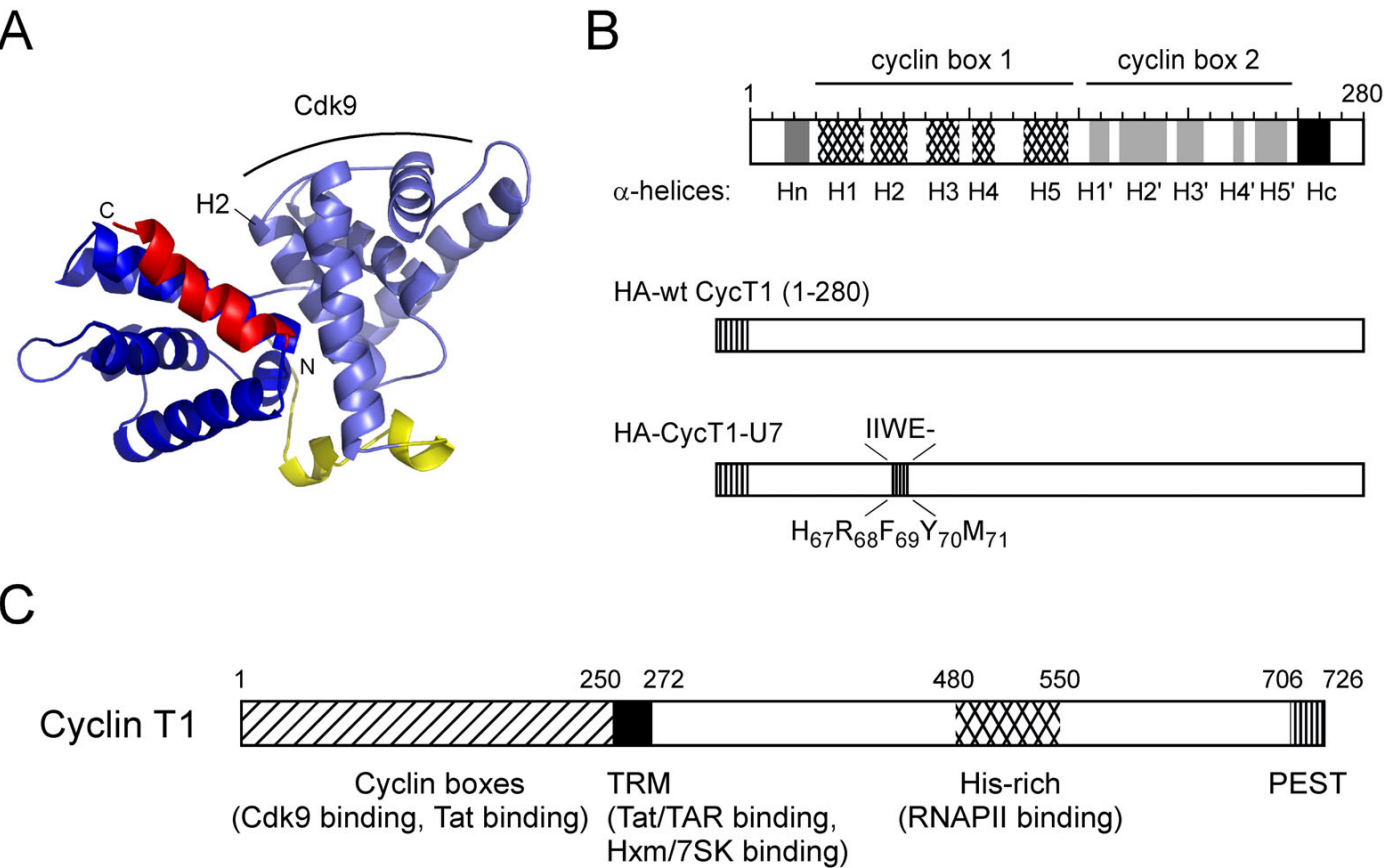
**Construction of mutant CycT1 proteins.A**. Structure of the cyclin box repeat domain (1–281) of CycT1. Two repeats of five α-helices each form the conserved cyclin box (blue). Flanking N- and C-terminal helices, which are important for the specificity of cyclins, are depicted in yellow and red, respectively. **B**. Schematic representation of C-terminally truncated wt CycT1 and the dominant negative CycT1-U7 mutant used in this study. Secondary structure of conserved α-helices (dotted regions in cyclin box 1 and hatched regions in cyclin box 2) together with two helices at N- and C-terminal (gray) locate in the N-terminal cyclin boxes in CycT1. Random mutations were introduced into the nine most conserved regions (shown by thin lines) in the cyclin box domain of a C-terminal truncation mutant of CycT1 (CycT1(1–280)). "-" in the CycT1-U7 sequence represents a deletion site. The truncated wt and mutant CycT1 employed in this study are also shown. **C**. A schematic representation of the full-length Cyclin T1. Amino acid motifs such as cyclin boxes, Tat-TAR recognition motif (TRM), coiled-coiled region, and PEST sequence are depicted.

CycT1 is a member of the C-type cyclin family [36]. Its N-terminal 250 amino acids form two cyclin repeat boxes that are essential for the interaction with, and the activation of, Cdk9. Recently, we have determined the three dimensional crystal structure of CycT1 [18]. The cyclin boxes consist of two repeats, each containing five α-helices (Figure. 1A and 1B). Sequence alignment of three P-TEFb-forming cyclins T1, T2, and K from different species revealed that the secondary structure elements are well conserved among these cyclins, indicating that they play important roles in P-TEFb functions (Figure. 1B). Based on this secondary structure alignment, we selected the nine most conserved amino acid clusters in the cyclin box domain of CycT1 and introduced random mutations into a C-terminal truncation mutant of CycT1 (CycT1(1–280)). This truncation is sufficient to support Tat transactivation as described before [4,5,9] (Figure. 1C and Table 1).

**Table 1:** Overview of CycT1 mutants used in this study.

Regions	Helix#	Amino acid positions	# of clones	Activity in murine cells (*1)		
				
				>100%	50–100%	<50%
Region 1	2	58–65	29	4	14	11
Region 2	2	67–71	26	3	10	13
Region 3	3	88–91	16	2	5	9
Region 4	3	93–96	16	3	11	2
Region 5	4	104–108	5	0	0	5
Region 6	5	132–137	3	0	1	2
Region 7	5	139–143	9	0	0	9
Region 8	5	145–148	2	0	0	2
Region 9	1'	149–152	9	3	4	2

total			115	15	45	55

*1: Tat transactivation obtained with wt human CycT1 is set as 100%

**Figure 2:**
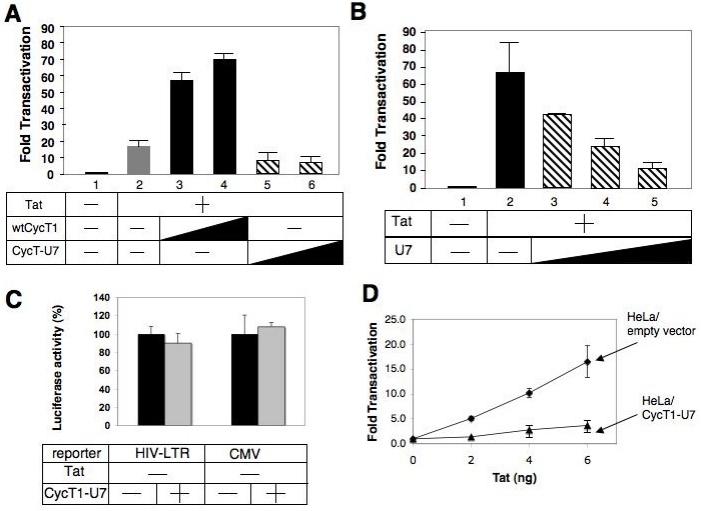
**N-terminal mutant CycT1 proteins (CycT1-U7) exhibit a strong dominant negative effect on HIV transactivation.A**. CycT1-U7 cannot support Tat transactivation in murine cells. NIH 3T3 cells were transfected with HIV-Luc reporter gene in the presence (lane 2–6) or absence (lane 1) of Tat (0.1 μg) with or without increasing amounts (0.2 and 0.5 μg) of wt human CycT 1–280 (lanes 3 and 4) or CycT1-U7 (lanes 5 and 6). Twenty-four hours after transfection, luciferase activities were measured as described before. **B**. CycT1-U7 shows strong dominant negative effects on Tat-transactivation. Increasing amounts of CycT1-U7 (0.2, 0.4 and 0.6 μg) were transfected in HeLa/pHR-Luc cells in the presence of Tat (0.02 μg). Luciferase activities were measured as described above. **C**. CycT1-U7 was unable to inhibit basal HIV transcription and CMV-driven transcription. The plasmid (0.6 μg) encoding CycT1-U7 (gray bars) or an empty vector (black bars) was co-transfected in HeLa cells with HIV-LTR-Luciferase or CMV-Luciferase reporter plasmid (0.05 μg) in the absence of Tat. Luciferase activity was measured as described above. **D**. Tat has lower activity on HIV-LTR in cells stably expressing CycT1-U7. Increasing amounts of Tat were transfected in Hela/pHR-Luc cells stably carrying a lentiviral vector encoding no protein (empty vector; gray diamonds) or CycT1-U7 proteins (black triangle). Luciferase activities were measured as described in the Materials and Methods section. Error bars represent the standard deviation of triplicate measurements. Data are representative of four independent assays.

Mutations were introduced by oligonucleotides containing degenerate nucleotides corresponding to each conserved region. In total, 115 CycT1 mutants were constructed and tested for their activities on Tat transactivation by co-transfecting murine NIH 3T3 cells with an HIV LTR-Luciferase (Luc) reporter gene and Tat (Table 1). Since murine endogenous CycT1 (mCycT1) cannot support Tat transactivation, Tat activated the LTR-driven Luc expression only by approximately 10-fold (Figure. 2A, lane 2). Over-expression of the wt human CycT1 further activated the gene expression up to 70-fold (Figure. 2A, lanes 3 and 4). The luciferase activities obtained by overexpressing any of the pool of mutant CycT1 proteins ranged from five to 70-fold. Fifteen mutants showed an equal or a higher activity than the wt, 45 mutants showed modest (50–100% of wt) activity and 55 had less than 50% of the activity of wt CycT1 in these cells (summarized in Table 1). These 55 mutants were further sequenced and tested for their dominant negative effect on HIV transcription by co-transfecting HeLa cells stably expressing the HIV-Luc reporter gene (HeLa/HR-Luc cells) with Tat (Figure. 2B and data not shown).

### An N-Terminal CycT1 mutant exhibited the strongest dominant negative effect on Tat transactivation by promoting the degradation of Tat proteins

**Figure 3:**
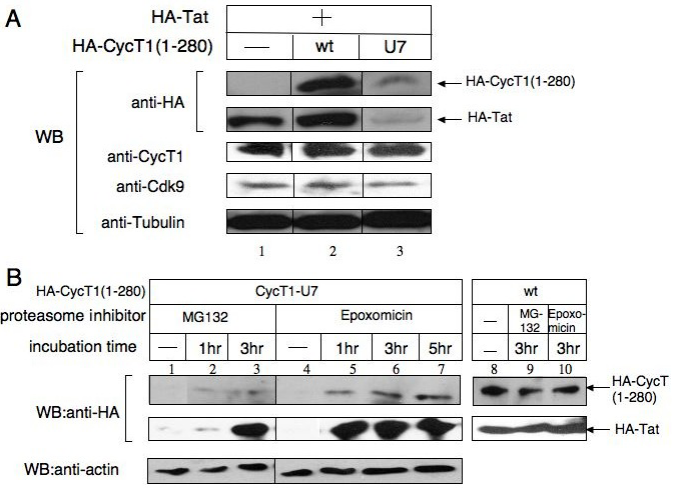
**CycT1-U7 promotes the degradation of Tat. A**. Western blotting depicts the steady-state expression of Tat proteins co-expressed with wt or mutant CycT1. HA-epitope tagged Tat (lanes 1–3) and HA-tagged CycT1 1–280 (wt: lane 2) or HA-CycT1-U7 (lane 3) were co-expressed in 293T cells. Twenty-four hours after transfection, cells were lysed with RIPA buffer (25 mM Hepes-KOH, 150 mM KCl, 1 mM EDTA, 1% Triton X100, 0.1% NP-40, pH 7.4), and soluble proteins were separated by 12% SDS-PAGE. The ectopically expressed CycT1 and Tat proteins were detected by anti-HA antibody. The endogenous proteins (CycT1, Cdk9 and Tubulin) were also detected by Western blotting. **B**. The expression of CycT1-U7 and Tat was restored by proteasome inhibitors. 293T cells were transfected with HA-tagged wt CycT1 (1–280) (lanes 8 to 10) or HA-CycT1-U7 (lanes 1 to 7) and HA-Tat as described above. Twenty-four hours after transfection, cells were treated with DMSO (lanes 1, 4 and 8), MG-132 (50 μM: lanes 2, 3 and 9) or Epoxomicin (50 μM: lanes 4 to 7 and 10) for 1 (lanes 2 and 5), 3 (lanes 3, 6, 9 and 10) and 5 hours (lane 7). Cells were then lysed in RIPA buffer and subjected to SDS-PAGE. The ectopically expressed CycT1 and Tat proteins were detected by anti-HA antibodies.

Amongst the 55 clones tested for their ability to block Tat transactivation in HeLa cells, one mutant containing four amino acid substitutions and one deletion in the second helix H2 of the N-terminal cyclin box repeat (residues HRFYM at a.a. position 67–71 to IIWE; Figure. 1B), termed CycT1-U7, showed the strongest dominant negative effect (>90% inhibition) on HIV transcription in HeLa/HR-Luc cells (Figure. 2B, lanes 3 to 5). At least four other mutant CycT1 constructed by the same oligonucleotides (Mut 2, Additional file 1) showed potent dominant negative effects on HIV transcription (60–90%, data not shown). Over-expression of CycT1-U7 affected neither the basal HIV transcription nor CMV-Luc reporter gene expression (Figure. 2C). Next, HeLa/HR-Luc cells stably expressing CycT1-U7 were created by infecting with a second lentiviral vector. Tat transactivation in these cells was scored by transfecting an increasing amount of Tat and measuring LTR-driven luciferase activity. In cells expressing CycT1-U7, Tat exhibited a significantly lower activity compared to the cells stably carrying the empty pHR lentiviral vector (Figure. 2D). Western blot analysis revealed that the steady-state expression level of CycT1-U7 is much lower than wt CycT1 (1–280) although the same amount of plasmid was transfected (Figure. 3A, lanes 2 and 3 in the top panel). Interestingly, the expression level of Tat was also much lower in CycT1-U7-expressing cells than in wt CycT1 (1–280) expressing cells, (Figure. 3A, lane 2 and 3). In contrast, the expression levels of the endogenous CycT1 and Cdk9 in the presence of CycT1-U7 remained unchanged (Figure. 3A). These results suggested that Tat transactivation in CycT1-U7 expressing cells is kept at a low level because the steady state Tat expression is diminished in these cells. Since CycT1-U7 retains the wild type sequence of Tat-TAR recognition motif (Figure. 1C), we hypothesized that CycT1-U7 forms a complex with Tat, and this complex is rapidly degraded in cells.

### Expression of CycT1-U7 and Tat can be rescued by proteasome inhibitors

To further prove our hypothesis that CycT1-U7, together with Tat, is rapidly transferred to proteasomal degradation pathways, cells expressing Tat and either wt CycT1 (1–280) or mutant CycT1-U7 were incubated with the proteasome inhibitors, MG-132 (50 μM) or Epoxomicin (50 μM) for 1, 3, and 5 hours prior to cell lysis. MG-132 showed a strong cytopathic effect when incubated for 5 hours (data not shown). The expression of both CycT1-U7 and Tat was partially restored in the presence of MG-132 (Figure. 3B, lanes 2 and 3 compared with lane 1), and much more efficiently restored in the presence of Epoxomicin (Figure. 3B, lanes 5 to 7 compared with lane 4). In contrast, the expression of wt CycT1 (1–280) and Tat remained virtually unchanged in the presence of these inhibitors (lanes 9 and 10 compared with lane 8).

**Figure 4:**
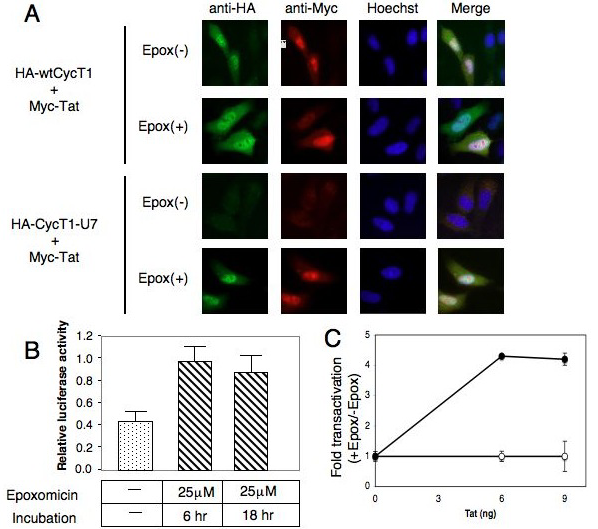
**Epoxomicin restores CycT1-U7 and Tat expression.A**. HA-tagged wt and mutant CycT1 and myc-tagged Tat proteins were co-expressed in HeLa/HR-Luc cells. Twenty-four hours after transfection, cells were untreated or treated with 25 μM Epoxomicin for 3 hours. HA-CycT1 proteins were visualized with mouse anti-HA antibody and Cy2-conjugated donkey anti-mouse IgG. Myc-Tat proteins were seen with Texas Red-conjugated anti-myc antibody. Nuclei were stained with Hoechst. **B**. The inhibitory effect by CycT1-U7 was diminished by Epoxomicin. HeLa/HR-Luc cells were transfected with CycT1-U7 expression plasmids (0.5 μg) or empty plasmids (0.5 μg) in the presence of Tat. Cells were treated with DMSO (-) or 25 μM Epoxomicin for 6 hours and 18 hours as indicated, and the Luc activities were measured. The results were presented as relative luciferase values obtained with CycT1-U7 divided by the values with the empty vector at each time point. **C**. Increasing amounts of Tat were transfected in Hela/pHR-Luc cells stably expressing CycT1-U7 proteins. 24 hours after transfection, cells were untreated (open circles) or incubated (closed circles) with Epoxomicin (25 μM) for 6 hours prior to luciferase assay. Data are presented as fold activation relative to the value obtained with untreated cells. Error bars represent the standard deviation of triplicate measurements. Data are representative of three independent assays.

**Figure 5:**
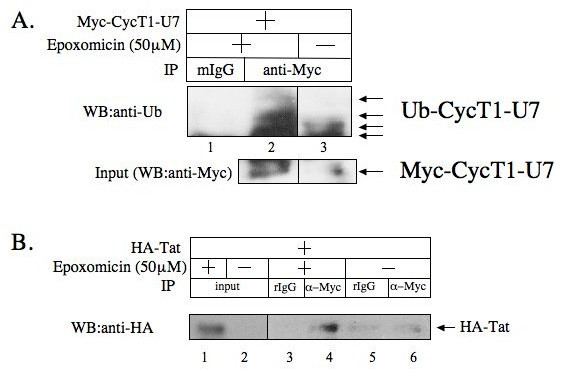
**CycT1-U7 is ubiquitinated.A**. HeLa cells stably expressing myc-CycT1-U7 proteins (HeLa/myc-CycT1-U7) were treated (lanes 1 and 2) or untreated (lane 3) with Epoxomicin (50 μM) for 30 min prior to cell lysis. The Myc-CycT1-U7 proteins were immunoprecipitated with anti-Myc antibody followed by Western blot analysis with anti-Ub antibody to detect ubiquitinated Myc-CycT1-U7 proteins (upper panel). Normal mouse IgG (mIgG) was used as a negative control for immunoprecipitation (lane 1). The expression of the Myc-CycT1-U7 proteins in 10% of the input samples was also detected by Western blot analysis using anti-Myc antibody (lower panel). **B**. CycT1-U7 binds Tat. HeLa/Myc-CycT1-U7 cells were transfected with HA-Tat. The cells were treated (lanes 1, 3 and 4) or untreated (lanes 2, 4 and 5) with Epoxomicin (50 μM) for 30 min prior to cell lysis. The Myc-CycT1-U7 proteins were immunoprecipitated with anti-Myc antibody (lanes 4 and 6) followed by Western blot analysis with anti-HA antibody to detect Tat proteins. Normal rabbit IgG (rIgG) was used as a negative control (lanes 3 and 5). Tat proteins in the input samples (10%) were also shown (lanes 1 and 2).

The restoration of the CycT1-U7 and Tat expression by Epoxomicin was also observed at the cellular level by an indirect immuno-fluorescence (IF) assay (Figure. 4A). HA-tagged wt and mutant CycT1 and myc-tagged Tat proteins were co-expressed in HeLa/HR-Luc cells. Twenty-four hours after transfection, cells were untreated or treated with 25 μM Epoxomicin for 3 hours. HA-CycT1 proteins were probed with mouse anti-HA and Cy2-conjugated anti-mouse IgG, and myc-Tat proteins were probed with Texas Red-labelled anti-myc antibody. As shown in Figure. 4A, the expression of CycT1-U7 and Tat was kept at low levels without Epoxomicin treatment. The protein levels were elevated when the cells were treated with Epoxomicin. The wt CycT1 and Tat proteins co-expressed with wt CycT1 were detected in the presence or absence of Epoxomicin. Finally, the inhibitory effect by CycT-U7 was diminished in transient (Figure. 4B) and stable (Figure. 4C) expression systems when the cells were incubated with 25 μM Epoxomicin for 6 to 18 hours. Since it has been demonstrated that CycT1 is ubiquitinated in cells [37], we sought to examine whether CycT1-U7 is ubiquitinated by co-immunoprecipitation analysis (Figure. 5A). Ubiquitinated CycT1-U7 proteins were detected in HeLa/CycT1-U7 cells treated with 50 μM Epoxomicin for 60 min (Figure. 5A, lane 2). Also, in this condition, the interaction between CycT1-U7 and Tat was detected by co-immunoprecipitation (Figure. 5B, lane 4). These results suggest that CycT1-U7 inhibits Tat-transactivation by rapidly recruiting Tat proteins into an ubiquitin-dependent proteasomal degradation pathway.

## Discussion

**Figure 6:**
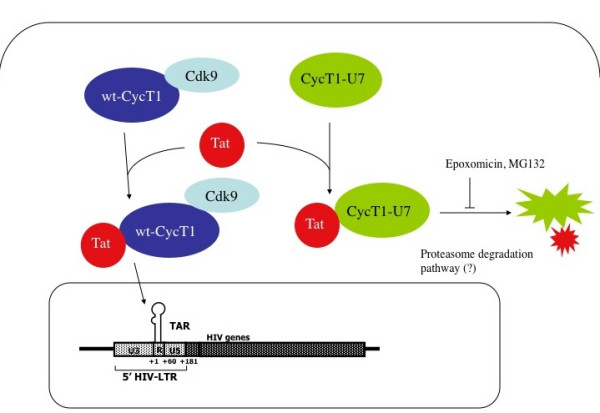
**Proposed model for the mechanism of dominant negative effect elicited by CycT1-U7.** Wild type CycT1 forms a complex with Cdk9 as an active P-TEFb, and interacts with Tat and TAR RNA. Alternatively, CycT1-U7 associates with Tat but the CycT-U7/Tat complex is immediately degraded *via *an ubiquitin-dependent proteasomal pathway. This degradation can be prevented using proteasome inhibitors.

Although P-TEFb is a potential target for the development of novel anti-HIV therapies, it had been extremely difficult to construct dominant negative CycT1 mutants that block HIV transcription [30]. This is presumably due to the high stability and the complex regulatory mechanism of the endogenous P-TEFb complex. In the present study, we constructed and evaluated a novel class of CycT1 mutant proteins (CycT1-U7) that explicitly block HIV transcription by promoting a rapid and specific degradation of Tat proteins co-expressing CycT1-U7. Resulting from a functional screen of 115 randomized mutant proteins, sequence analysis of CycT1-U7 showed five mutations including one amino acid deletion in the second helix of the cyclin box repeat fold (Figure. 1B). We have previously demonstrated that a CycT1 variant lacking this region (CycT1 (119–280)) is also unstable in cells [38]. This particular mutant exhibited a potent dominant negative effect on HIV transcription, potentially by a similar mechanism (data not shown). Therefore, H2 of CycT1 appears very important for maintaining the structural stability of CycT1 and the interface in between the two repeats. In addition, a residue directly preceding the first helix of the cyclin box repeat that varies between human and equine CycT1 has been previously identified as responsible for differences in the recognition of Tat/TAR complexes from HIV and EIAV [39]. Together, these data point towards the importance of the integrity of the first cyclin box repeat for the interaction with Tat. This region also appears to be essential for the interaction with Cdk9 [18,30]. Interestingly, CycT1-U7 does not promote degradation of endogenous Cdk9. On the other hand, this mutant does bear the wild type sequence of Tat/TAR recognition motif (a.a. 251–272). Indeed, the complex between CycT1-U7 and Tat was detected when the cells were treated with proteasome inhibitors (Figure. 5). The mutant CycT1-U7 proteins can form a complex with Tat and this complex would be immediately degraded because of the instability of CycT1-U7. Therefore, we conclude that CycT1-U7 exhibits a strong dominant negative effect on Tat transactivation by specifically degrading the co-expressed Tat protein, without disturbing the endogenous P-TEFb complex (Figure. 6).

It has been demonstrated that CycT1 interacts with other cellular transcription factors through its N-terminal cyclin box regions [40,41]. It is of importance to examine whether CycT-U7 can also inhibit cellular transcription mediated by these factors *via *a similar pathway. Additionally, the TRM region of CycT1 also interacts with HEXIM1, the endogenous inhibitory protein of P-TEFb which interacts with this region [11], and it is possible that CycT1-U7 affects P-TEFb activity by reducing HEXIM1 levels. More detailed studies are required to assess the effect of CycT1-U7 on cellular transcription.

Our results indicate that CycT1-U7/Tat is recruited to the ubiquitin-dependent degradation pathway. CycT1 seems to be ubiquitinated not only on its C-terminal PEST region (a.a. 706–726) but also at other regions [37]. It is to be noted that wt CycT1 (1–280) is resistant to degradation (Figure. 3). Although we have not identified the potential ubiquitination site(s) of CycT1-U7 in this study, it is possible that the cyclin box structure stabilizes the protein by preventing ubiquitination. Conformational changes induced by post-translational modifications such as phosphorylation may expose any additional ubiquitination sites in this region, which would represent a novel pathway to regulate P-TEFb function.

Constructing CycT1 mutants based on C-terminal truncated forms of wt CycT1 (CycT1(1–280)) is particularly beneficial in terms of HIV transcription. CycT1 (1–280) has been demonstrated to be sufficient for supporting Tat-transactivation [4,5,9]. In addition, Tat competes with HEXIM1 to increase active P-TEFb complexes [14,15,19]. CycT1 (1–280) can therefore bypass the 7SK/HEXIM-mediated complex regulatory pathway and be exclusively directed towards Tat-dependent transactivation (Jadlowsky et al., unpublished data), making CycT1 (1–280) proteins highly specific for Tat.

Since the mechanism by which CycT1-U7 inhibits HIV transcription seems not to be through blocking the normal function of P-TEFb, but rather through a "gain-of-function" pathway, it represents a novel class of inhibitory molecules. Moreover, since the steady-state expression of CycT-U7 is very low, it may be an excellent candidate for gene therapy because the mutant proteins would not persist for a prolonged period of time, thereby avoiding induction of unwanted immune responses. Additionally, these proteins would work only when Tat is actively expressed in cells.

HIV utilizes the cellular transcriptional machinery for its own replication. Therefore, it is important to inhibit this step without disturbing cellular functions. Since CycT1 interacts with Tat and TAR, it can be an excellent target to develop safe and effective anti-HIV therapies. Here we present an example of a dominant negative CycT1 molecule which specifically blocks HIV transcription. Studying the precise mechanism by which this mutant CycT1 protein inhibits HIV transcription could unveil novel regulatory pathways of the HIV life cycle and therefore provide reliable clues for designing anti-HIV agents.

## Conclusion

In this study, we constructed and evaluated dominant negative CycT1 mutant proteins that specifically block HIV transcription by promoting a rapid degradation of Tat proteins. These mutant CycT1 proteins represent a novel class of specific inhibitors for HIV transcription, which can be further utilized to develop a safe and effective anti-HIV therapy.

## Methods

### Materials

HeLa, 293T or NIH 3T3 cells were maintained in Dulbecco's Modified Eagle's Medium (DMEM) including 10% fetal bovine serum at 37°C with 5% CO_2_. HeLa cells stably carrying an HIV-LTR-driven luciferase reporter gene (HeLa-HR-Luc cells) were established using pHR lentiviral vector expressing the luciferase gene under the control of the HIV-LTR, as described previously [42,43]. Anti-myc, anti-HA, anti-CycT1, anti-Cdk9, and anti-Ub antibodies were purchased from Santa Cruz Biotechnology (Santa Cruz, CA). Anti-actin antibody was purchased from Cell Signaling Technology (Danvers, MA). Anti-Tubulin was purchased from Sigma Aldrich (St. Louis, MO). Proteasome inhibitors, MG-132 and Epoxomicin were purchased from EMD Bioscience (San Diego, CA) and Alexis (San Diego, CA), respectively.

### Construction of CycT1 mutants

A structure-based sequence alignment resulting from the crystal structure of the cyclin box repeat of human CycT1 [18] revealed highly conserved α-helical structures in the P-TEFb-forming cyclins T1, T2 and K (Figure. 1B). Based on this alignment, we selected the nine most conserved regions in the cyclin box repeat domain of CycT1 and introduced random mutations into a C-terminal truncation mutant of CycT1 (1–280) by using oligonucleotides that contain degenerated nucleotides at positions corresponding to each conserved helix and the Transformer Site Directed Mutagenesis Kit (Clontech) (Figure. 1 and Table 1). The resulting 115 CycT1 mutants were tested for their ability to support Tat transactivation in murine cells as described previously [4]. The CycT1 mutants that failed to activate HIV-transcription in murine cells were sequenced and further tested for their ability to block Tat transactivation in HeLa cells as described previously [30]. The mutant CycT1 (termed CycT1-U7) that exhibited the strongest inhibitory effect on Tat-dependent HIV transcription was used in this study. Sequences of the mutagenic oligonucleotides are shown in Additional file 1.

### Generation of stable cell lines

CycT-U7 was subcloned downstream of a CMV promoter in a modified pHR'-SIN lentiviral vector [44,45]. The VSV-G pseudotyped lentiviruses were produced by co-transfection with packaging plasmids (pMDG and p8.9I, [46]), and used to infect Hela cells and HeLa-HR Luc cells.

### Transfection and reporter assays

HeLa or NIH 3T3 cells were transfected with 0.5 μg of pEF-CycT1 (wt or mutant constructs) and an HIV-Luciferase reporter construct, in the presence or absence of pTat (0.01 μg) using Lipofectamine 2000 according to the manufacturer's instructions (Invitrogen). Twenty-four hours after transfection, cells were harvested and lysed. The protein concentrations of the cell lysates were determined by Protein Assay kit (BioRad). Luciferase activities in the cell lysates were measured as described previously [43].

### Ubiquitination assays

HeLa cells stably expressing myc-epitope tagged mutant CycT1 proteins were expressed and, when indicated, treated with 50 μM Epoxomicin for 1 hour. Cells were lysed in radio-immunoprecipitation assay (RIPA) buffer (50 mM Tris-HCl, 0.15 M NaCl, 1 mM EDTA, 1% Sodium deoxycholate, 1% NP-40, 0.1% SDS, 1 mM DTT [pH 7.4]) in the presence of protease inhibitors. After preclearing with protein-G sepharose coupled with normal mouse IgG, cell lysates were incubated with 0.5 μg of monoclonal antibody against c-Myc (F-7; Santa Cruz Biotechnology) overnight at 4°C. After the cell lysates were allowed to bind to the antibody, reaction mixtures were incubated with protein-G sepharose beads (Roche) for 1 hour at 4°C. The beads were washed extensively with RIPA buffer and the proteins remaining on the beads were eluted by incubation with SDS loading buffer (50 mM Tris-HCl, 2% SDS, 10% glycerol, 2 mM EDTA, 0.1 M DTT and 0.01% bromophenol blue, pH 6.8) and subjected to SDS-PAGE, followed by Western blotting with anti-Ub antibody (Santa Cruz Biotechnology).

### Proteasome inhibitor treatment

293T cells (2 × 10^5^) were transfected with 1 μg of plasmids encoding HA-tagged wt CycT1 (1–280) or CycT1-U7 in the presence or absence of the plasmid encoding HA-tagged HIV-1 Tat (0.2 μg) using calcium phosphate. Twenty-four hours post-transfection, cells were treated with MG-132 (50 μM), Epoxomicin (50 μM) or DMSO (solvent control) at 37°C for 1, 3 and 5 hours. Cells were then harvested and lysed in RIPA buffer. The protein concentrations in the cell lysates were determined by Protein Assay Kits (BioRad, Palo Alto, CA). The same amounts of cellular proteins (20 μg) were separated by SDS-PAGE followed by Western blot analysis to detect the HA-epitope tagged CycT1 proteins and myc-epitope tagged Tat proteins.

### Immunofluorescence (IF) assay

HA-tagged wt and mutant CycT1 and myc-tagged Tat proteins were co-expressed in HeLa/HR-Luc cells using Lipofectin (Invitrogen). Twenty-four hours after transfection, cells were untreated or treated with 25 μM Epoxomicin for 3 hours. Cells were fixed with 4% formaldehyde and blocked with phosphate-buffered saline (PBS) containing 0.1% Triton X-100, 1% sodium azide and 10% normal donkey serum. After washing with PBS, HA-CycT1 and were probed with mouse anti-HA monoclonal antibody (Santa Cruz) and Cy2-conjugated donkey anti-mouse IgG (Jackson ImmunoResearch). Myc-Tat proteins were probed with anti-Myc monoclonal antibody (Santa Cruz) pre-labeled with Zennon ^® ^Texas Red ^® ^anti-Mouse IgG1 (Invitrogen). Nuclei were stained with Hoechst (Sigma). Fixed cell images were captured on a Deltavision DV-RT (Applied Precision, Inc. Issaquah, WA.) microscopy system using the Deltavision Softworx program.

## Competing interests

The authors declare that they have no competing interests.

## Authors' contributions

JKJ carried out luciferase assays and protein assays, participated in designing the experiments and drafted the manuscript. NM constructed the mutant proteins. AS participated in the structural analysis of CycT1 protein. MG participated in sequence alignment and designing the mutant protein. TO participated in the prediction of mutant protein structure. KF participated in designing the experiments, performed biochemical experiments and helped to draft the manuscript. All authors read and approved the final manuscript.

## Supplementary Material

Additional file 1 Sequences of the mutagenic oligonucleotides. Click here for file
