# Supplementary material for: Dominant negative mutant Cyclin T1 proteins inhibit HIV transcription by specifically degrading Tat
Source: Retrovirology. 2008 Jul 11;5:63. doi: 10.1186/1742-4690-5-63 (PMC2492875; doi:10.1186/1742-4690-5-63)
Supplement: Additional file 1 — Sequences of the mutagenic oligonucleotides. [file 1742-4690-5-63-S1.doc]

| Name | Sequence | Position(a.a) |
| --- | --- | --- |
| Mut-1 | GTCTCACAATTGNNNATCAACNNNGCTATAGTANNNATGCATCGATTC | 58-65 |
| Mut-2 | CACTGCTATAGTATACATGNNNNNNNNNNNNNNNATYCAGTCCTTCACAC | 67-71 |
| Mut-3 | GCTCCAGCAGCCNNNNNNNNNNNNGCTAAAGTGGAGGAGCAGCCC | 88-91 |
| Mut-4 | GCTAAAGTGGAGGAGNNNNNNNNNNNNNNNTTGGAACATGTCATCAAGGTAGC | 93-96 |
| Mut-5 | CAAAAAATTGGAACATNNNNNNNNNNNNGCANNNNNNTGTCTCCATCCTC | 104-108 |
| Mut-6 | TTGCAACAAGTTCAANNNNNNNNNNNNNNNNNNAGCATAATTTTGCAGTT | 132-137 |
| Mut-7 | CTGGTCATTTTAAGNNNNNNNNNNNNNNNNNNTTAGGCTTTGAACTAAC | 139-143 |
| Mut-8 | ATTTTGCAGACTTTAGGCNNNNNNNNNNNNATTGATCACCCACATACTC | 145-148 |
| Mut-9 | CTTTAGGCTTTGAACTANNNNNNNNNNNNNNNCATGTAGTAAAGTGCAC | 149-152 |
|  |  |  |
|  | N=A,T,G or C |  |

Sequences of the mutagenic oligonucleotides.
